# When are you dead enough to be a donor? Can any feasible protocol for the determination of death on circulatory criteria respect the dead donor rule?

**DOI:** 10.1007/s11017-019-09500-0

**Published:** 2019-09-28

**Authors:** Govert den Hartogh

**Affiliations:** Department of Philosophy, University of Amsterdam, Amsterdam, Netherlands

**Keywords:** Definition of death, Determination of death, Brain death, Circulatory death, Dead donor rule, Irreversibility, Legal fiction

## Abstract

The basic question concerning the compatibility of donation after circulatory death (DCD) protocols with the dead donor rule is whether such protocols can guarantee that the loss of relevant biological functions is truly irreversible. Which functions are the relevant ones? I argue that the answer to this question can be derived neither from a proper understanding of the meaning of the term “death” nor from a proper understanding of the nature of death as a biological phenomenon. The concept of death can be made fully determinate only by stipulation. I propose to focus on the irreversible loss of the capacity for consciousness and the capacity for spontaneous breathing. Having accepted that proposal, the meaning of “irreversibility” need not be twisted in order to claim that DCD protocols can guarantee that the loss of these functions is irreversible. And this guarantee does not mean that reversing that loss is either conceptually impossible or known to be impossible with absolute certainty.

## The problem

To put the point paradoxically, the traditional “brain-dead” donor is slowly dying out [1]. The main reason for this is steady improvement in the possibilities for treating subarachnoid hemorrhage, by itself a happy circumstance. In many countries, road safety has been enhanced as well. Also improving are techniques for transplanting organs from DCD donors—where “DCD” stands for donation after circulatory (rather than cardiac) death.^1^ As a result, the prognosis for the length and quality of life of the recipient by now is comparable to the prognosis in the case of donation after brain death (DBD), at least as regards kidney and heart transplantation [2, 3]. Hence in an increasing number of countries, the percentage of DCD donations is growing [4].

Of course, in many cases a potential DCD donor can eventually become a DBD donor if mechanical ventilation is prolonged until the criteria for total brain failure have been met. This is what usually happens in some countries—France and Spain, for example.^2^ As a result, DCD in these countries occurs only after failed attempts at reanimation in cases of unexpected cessation of respiration (so-called uncontrolled or unexpected DCD). In other countries, at a certain point treatment is considered futile; hence the treating team is supposed to have the moral and legal obligation to stop it. (Or a legal representative for the patient may make a binding request to stop treatment.) In some cases, brain death may be suspected, but the family will consent to organ retrieval only after asystole, since this satisfies their need to witness an observable ending of life [6].

When the ventilator is turned off, after a while warm ischemia sets in, marking a deprivation of sufficient nourishing blood and oxygen with damaging effects on the organs. Hence DCD protocols provide for a (widely varying) maximum period of time between the withdrawal of treatment and the determination of death by circulatory criteria. But from that last moment on, the deterioration of the organs accelerates, so it is imperative to start removing them as quickly as possible. It would probably be impossible,^3^ and in any case would take too much time, to investigate whether the criteria for total brain failure are satisfied at that point, but it is supposed that after some minutes these will have been met anyway as a result of ischemic damage. Many protocols require a waiting period of five minutes,^4^ but some American protocols require only two minutes.

Why is it supposed to be so important to wait? The reason is the dead donor rule (DDR), which stipulates that one should not start removing a person’s organs before she is dead. The DDR is usually seen both as a fundamental moral principle and as a necessary bulwark for the protection of public trust; and although it is explicitly stated only in some relevant statutes, such as certain Canadian ones, it is always implied by other provisions, in transplantation and even homicide law. A donation law may, for example, stipulate that death has to be determined by a physician who is in no way associated with the removal or the implantation of organs (e.g., [9], art. 14 lid 1).

Since the (re)introduction of DCD in the beginning of the nineties, critics have alleged that, regardless of the waiting period, DCD necessarily violates the DDR, and recent medical developments have only served to fuel their criticism. Currently this seems to be the dominant view in the bioethical literature. If it is true that DCD necessarily violates the DDR, then one is confronted with an unattractive dilemma: either stop DCD (or go on forbidding it, as professional medical rules in Germany do) or abandon the DDR. A third option would be to go on hypocritically paying lip service to the DDR while violating it in practice, which is what critics suggest is happening at present. My aim in this paper is to evaluate this criticism.

I cannot discuss whether it is really all that important to hold on to the DDR, but I will presuppose that it is. My own view is that the DDR is not a fundamental moral truth. Indeed, I believe that in a pluralistic society, the law should allow people to declare that the DDR does not apply in their case.^5^ Allowing this choice would respect individual autonomy and, if sufficient physicians are prepared to act on such declarations, result in the availability of more organs of better quality. It is more or less generally agreed, however, that surrendering the DDR would indeed involve risks to the maintenance of public trust, although it is difficult to estimate the size of those risks on the available evidence.^6^ Even if one is inclined to believe that, at least in the long run, abandoning the DDR might not have the catastrophic impact on donation rates that politicians and doctors fear, this seems a dangerous experiment to try out. If one really were to face the dilemma (or trilemma) I just described, one should probably choose to abandon the rule, but one should not do so as long as such a choice can be avoided.

The first issue I address is the vexed problem of the proper criteria for the determination of death. I argue that such criteria cannot be derived from the correct “definition of death,” but rather the best proposal on the table consists of two necessary and jointly sufficient conditions: the irreversible cessation of consciousness and the irreversible loss of the capacity for spontaneous breathing [12].^7^ This proposal resembles the traditional British view [15], defended long ago by Bryan Jennett [19] and Christopher Pallis [12, 13] and adopted in a 2008 white paper by the majority of the President’s Council on Bioethics [17]. It differs from that view, however, insofar as I argue that these standards should not be understood as elements of a conception of either brain death or brainstem death.

The second and main issue I address is, then, whether any feasible DCD protocol can respect the DDR if the DDR is operationalized in terms of the aforementioned criteria for the determination of death. To the extent that the criticisms of bioethicists have been considered in guidelines and position statements at all, they have not been dealt with in a satisfactory way. My question is whether it is possible to do better. What I want to explore in particular is one way, possibly the only way, to sail through the horns of the dilemma, saving both DCD and the DDR.

## Standards for the determination of death

When is it appropriate to declare someone dead? This is usually referred to as the problem of the “definition” of death. I shall avoid that description, however, because it often conflates two different questions: the semantic question of which conditions are necessary and sufficient for applying the term “dead” to any entity in general, or to human beings in particular; and the theoretical question of what is common to all dead things, or all dead people, by virtue of which they are dead. This latter question is metaphysical, not biological, though any plausible answer to it has to be thoroughly biologically informed. It concerns the essential characteristics distinguishing important kinds of things in the world from one another.

The term “death” has, indeed, some obvious connotations that restrict its possible application, particularly when it refers to people. I suggest that two such necessary conditions can be agreed upon. First, a person cannot be said to be dead when she still has any vestiges of consciousness—whether or not she has to be ventilated permanently in order to retain such vestiges. A person with a high-level spinal cord injury may be unable to breathe without artificial support, but may nevertheless retain full consciousness. No one would call her dead. The same is true of any other conscious person with respiratory failure. Second, whatever one has to lose in order to be declared dead (including consciousness), one has to lose it irreversibly. (I will consider this tricky concept later on.) These are clearly only necessary conditions. The problem is that if one tries to go beyond them to formulate a set of separately necessary and jointly sufficient conditions, one gets mired in endless dispute, as the history of the debate over the last half-century shows.

If there has ever been anything approaching consensus, it did not last for more than a few years. In its internationally authoritative 1981 report on *Defining Death*, the President’s Commission for the Study of Ethical Problems in Medicine and Biomedical and Behavioral Research made the following proposal: “An individual who has sustained either (1) irreversible cessation of circulatory and respiratory functions, or (2) irreversible cessation of all functions of the entire brain, including the brain stem, is dead” [20, p. 2]. These criteria were not supposed to be on the same level. The metaphysical view of death that underlies the report is that life is a matter of the integrated functioning of major organ systems. The brain, however, is not merely one of those organ systems; according to the report, it directs the functioning of the other systems. Hence the irreversible cessation of circulatory and respiratory functions can be accepted as a criterion for the determination of death (in the absence of mechanical support of these functions) only because this is seen to *prove* the irreversible cessation of all functions of the entire brain, and thereby the integrated functioning of the organism. By applying the circulatory criterion, one could assess indirectly whether the neurological criterion has been satisfied: it is a mere diagnostic standard. The neurological standard, on the other hand, is presented as the basic one, the necessary and sufficient condition for a person to be dead.^8^

At the time, the consensus on this view, though never complete, was impressive. But doubts reemerged quickly. On the view of the President’s Commission, the brain was the all-important organ to consider because it was supposed to exercise control over all other biological functions, securing the functioning of all parts of the organism as an integrated whole [20, p. 33; 21; 23]. This view has been extensively criticized, in particular by Alan Shewmon [24–26] (well summarized in [17]). A list of biological functions that have been observed in brain-dead but mechanically ventilated people includes: maintenance of some degree of hemodynamic stability and body temperature, assimilation of nutrients, detoxification and excretion of wastes, wound healing, immune response to infection, fever, demonstration of a stress response to incisions made for organ retrieval, proportional growth, sexual maturation, lacrimation, and even gestation of fetuses to viability. Some of these functions are conditions for the continuation of others, including brain functions, and can therefore be said to play a role in the organization of the organism as an integrated whole, a property the President’s Commission thought to be possessed merely by brain functions. If this criticism is accepted,^9^ the integration of bodily functions appears to be only partially due to brain activity; no single bodily structure plays the role of indispensable integrator. As the President’s Council on Bioethics puts it in their influential 2008 white paper, integration “is an emergent property of the whole organism—a property that does not depend upon directions from any one part, but is the product of the orchestration of multiple parts” [17, p. 40].

But if brain functions are not necessarily privileged, which other functions should we focus on, and why?

We could try to decide this by intuition, but the problem is that people’s intuitions are divergent, and each often ambiguous to boot. The critics of the whole-brain definition of death point to Shewmon’s impressive list of biological functions and ask, rhetorically, if an individual on a mechanical ventilator could go on exhibiting all or many of these functions for months,^10^ how can one possibly say that he is not alive? Well, others say, equally rhetorically, one may suppose these functions (or some of them) to occur in a decapitated body that is somehow mechanically ventilated, but would one call such a decapitated human being alive for that reason [13, p. 4; 20, p. 36; 30, pp. 28, 107, 393; 31]? Maybe because of all those ongoing functions, the decapitated body is not exactly a mere corpse through which air and blood are being pumped, but he is still something very close to it—a kind of living organism–machine hybrid. Or perhaps it should be granted that the decapitated body is a living entity with human DNA in the nucleus. But so is a chunk of human cancer cells growing in a culture or a hydatidiform mole. Surely the decapitated body shows more integration than a mole, but it is questionable whether it shows enough integration to be called a living human animal. As Shewmon himself has observed, there is no validated measuring scale for degrees of integration, and even if there were, there would be no way to nonarbitrarily identify on that scale the dividing line between dying and dead organisms [32, p. 259] (cf. [33], quoted in [18, p. 85]). It is true that if someone sees a warm and breathing body, possibly feverish in response to infection, exhibiting limb movements in response to stimuli, wearing diapers and catheters that need to be changed, she would be inclined to consider it alive, even if there is no sign of consciousness. But if she is told that the body is being kept in this condition only by mechanical ventilation, she might start to hesitate.^11^

The problem is not a matter of lacking knowledge, as the authors of the white paper and others suppose (e.g., [35, ch. 6; 36; 37). The reason for this supposition seems to be the idea that death is a biological event. I agree. But it does not follow that biological data can identify *which* event. Empirically, a list of functions that do not all stop at the same time can be established, and these stoppings do not necessarily take the same amount of time to become irreversible. The question, then, is which of these functions, or combination thereof, is *essential* for life—a question that no amount of new data can answer.

So it must be concluded that the concept of death is *indeterminate*: if in applying the concept of death, we use the criteria that are commonly recognized as appropriate, we do not get a determinate result in all cases. “Death is a fuzzy set” [38, p. 72] (cf. [39; 40, pp. 219–232]).^12^ In such cases, the facts, and not merely the available knowledge of the facts, underdetermine the application of the concept. Of course, one is either alive or dead; there is no further possibility. And because death is a binary concept, one cannot be more dead or less dead either, in spite of what the title of this article suggests. But it does not follow, as the white paper suggests, that if it cannot be determined whether one is alive or dead, the only possible reason is a lack of sufficient knowledge. Many concepts have a zone of indeterminacy; there is nothing special about this—go into the park and try to identify what vegetable object is a tree and what is a mere bush. And it is no wonder that our common concept of death is indeterminate. We have had it for ages without having to tailor it to the possibility of maintaining biological functions by mechanical ventilation.

## Choosing standards

Now we cannot leave the matter at that: people have to fix on some moment within the zone of indeterminacy to start changing their behavior. They have to know when to start mourning or when to grant rights to insurance; even the identification of a heir could depend on it. Without an identifiable moment of death, even the DDR loses its meaning. So how are we to proceed? If an appropriate moment cannot be derived from the meaning of the term “death” because its denotation is not determinate enough, we can proceed only by making a stipulation. That stipulation, however, need not be arbitrary. It can be argued for in two ways.

First, one can appeal to a metaphysical account of the nature of death. One such account has been noted already: the proposal of the President’s Commission to count an organism as alive so long as it functions as an integrated whole. This account turns out not to support a whole-brain definition of death, but it does succeed in explaining why an organism can be dead when some of its parts are still alive.

The problem with this account, however, is that it does not explain how much integration is required, and therefore does not help to sufficiently narrow down the zone of indeterminacy. A brain-dead body on life support is a collection of living cells, organs, and tissues that can coordinate with each other to some limited extent, but it may still lack the genuine integration that characterizes a living human organism.^13^

A second theoretical account starts from the strong and common intuitive belief that an individual who is spontaneously breathing, without any mechanical support, is alive. This intuition can be explained by pointing out that it is part of the traditional connotation of being alive that a living thing has the property of movement in itself. In modern terms, it is able to organize its own resistance to entropy. As the white paper elaborates, the fundamental vital work of a living organism is the work of the organism’s self-preservation, achieved through its need-driven commerce with the outside world [17, p. 60]. This work requires receptivity to relevant stimuli and an adequate response, both prompted by an inner drive [17, p. 61]. It could be added that the work of self-preservation also requires that the organism’s various metabolic processes function in an integrated fashion, maintaining homeostasis. The two accounts should therefore not be seen as rivals.^14^

The problem with this second account is that it does not explain the first necessary condition for death laid out above—namely, that a person cannot be dead if she has not lost the capacity for consciousness. If it were essential for an organism’s being alive that all of its functions be supported by its own appetitive nature and not by external mechanisms, at least not permanently, we would not be licensed to regard as alive the individual with a high-level spinal cord injury who must rely on mechanical ventilation in order to continue his possibly unimpaired mental activity. True, his consciousness enables him to engage in major forms of “commerce with the world,” but this commerce cannot be “self-driven.” He is no longer able to organize his own resistance to entropy without assistance; he has hardly more “integrative unity” than an individual with total brain failure [50]. Of course, opponents of the total brain failure criterion would argue: if it is not necessary for all functions to be unsupported in this case, then why is it necessary in others?^15^ For example, cardiac activity can take place without any direction from the brain, provided that respiration also takes place. So why is consciousness allowed to be permanently dependent on mechanically induced respiration, but cardiac activity is not? The mere fact of such support cannot mean that an individual is no longer alive.^16^

That argument, however, does not prove that the fact of support is always irrelevant. If someone still has the capacity for consciousness, she still exists as an embodied mind, even if that mind is embodied in an organism that is unable to sustain itself without artificial support. So how can we doubt whether the same organism—think again of the patient with a high-level spinal cord injury, or think of a patient dependent on a dual-chamber pacemaker—is alive when that organism is no longer able to embody a mind? It is precisely for that reason: because, in addition to losing the capacity for unsupported respiration and ventilation, the organism has lost the capacity, supported or unsupported, to uphold conscious experience. This capacity for conscious experience is on all accounts a biological function of special importance.

The idea that a living organism is characterized by its ability to maintain homeostasis in its internal environment on its own, and thereby withstand entropy, is plausible, but this idea is not enough for deciding how much support is compatible with the ascription of that ability to an organism. The appeal to theoretical considerations, therefore, cannot on its own provide the determinacy required. So it becomes necessary to turn to the second possible way of achieving determinacy: appeal to normative considerations [40, p. 232; 43, p. 27]. One can properly ask when the law is justified in *counting* a person as dead. Why is it appropriate to allow consciousness to be dependent on mechanically induced respiration, but not on cardiac activity? The answer is that we care for conscious states, but not for cardiac activity as such.

What is so valuable to us, or to some of us, that it requires protection by the DDR? Many people would like to avoid having their organs taken out when they are still conscious, or could return to a state of consciousness. If that were all people cared about, then the focus should be not on death as the salient dividing line, but on the irreversible loss of consciousness as such, preferably without troubling the waters by calling this “death.”^17^ A number of people, in addition, would like to avoid having their organs taken out when they still have the capacity for spontaneous breathing. But few people would like to keep being ventilated mechanically when both the capacity to have mental states and the capacity for spontaneous breathing have been lost. These few people would not consent to organ donation anyway.^18^

For these reasons, it seems reasonable to propose use of the following criteria for the determination of death: it requires irreversible loss of the capacity for consciousness and irreversible loss of the capacity for spontaneous respiration.^19^ This is—with a difference to be noted below—the traditional British view of Jennett and Pallis [12, 13, 19], now adopted by the 2008 white paper (the majority view) and still endorsed by the 2013 British guidelines for DCD [61]. On these criteria I agree with the position of the white paper. But on two points I disagree with its British approach.

First, I do not think of these two criteria as elements of a true conception of the nature of death, contra Jennett and Pallis. Because the facts, and not merely one’s understanding of the facts, underdetermine the application of the concept of death, the determinacy needed for the use of the DDR can be achieved only in a stipulative way, by appealing to normative considerations—in particular, to the rationale of the DDR itself. Is it not disingenuous by itself, or even paternalistic, to let the general public believe that the determination of death is purely a matter of medical expertise, while it is in fact to some limited extent informed by normative considerations [48, 62]? Surely it is not uncommon for cases in which ethics and law require informed consent that the information to be provided can be only a simplified version of a highly complicated truth [63]. And even if my analysis in this section were to be undisputed, one could hardly expect the general public or even the medical community at large to take the time to study and understand it. If such simplifications were rejected altogether, relevant decisions would be allowed to be made almost standardly on the basis of predictably mistaken fears and hopes, and hence would not be “informed” in any meaningful sense at all. Thus, if one were somehow to succeed in changing the law—defining “death” in terms of ongoing respiratory–circulatory functions, supported or not, and abandoning the DDR—one would not thereby improve the average person’s autonomous decision-making by giving her an extended menu of reasonably well-understood options. Yes, normative judgments are unavoidably involved in the use of the concept of death by doctors and courts, but it would be imperative to insist that this fact be fully understood in all cases only if these judgments would be the object of fundamental controversy, such that disclosure of their normativity could therefore be expected to lead some people, having fully understood this, to deviate from these judgments in a rational way.

The second point on which I disagree with the view of the white paper is as follows: in going British, its authors did not fully realize how revolutionary their proposed break with orthodox thinking about death would be. They understood their argument as a defense of a neurological standard of death. After all, both the capacity for consciousness and the ability to breathe spontaneously depend on the ongoing functioning of the brain. This view, however, fails to distinguish between basic and diagnostic criteria. In the 1981 report of the President’s Commission, as seen above, the basic standard for the determination of death is the total cessation of all brain functions. The report also allows a patient to be considered dead when respiration and circulation have stopped, but only because this is seen as a *sign* of death in the basic sense. For if the cessation of respiration and circulation is not the result of total brain failure, it always quickly leads to it. In 2008 this order is, in effect, reversed. By then, irreversible cessation of spontaneous respiratory and circulatory function is, in addition to the irreversible cessation of consciousness, a necessary condition of death—hence a basic standard. Total brain failure, however, is relevant only because it either is already caused by the cessation of spontaneous breathing or will cause it. Hence it is supposed to be only a reliable *sign* that one of the basic conditions of death has been met. That sign must be relied on when, on account of the presence of a ventilator, it cannot be directly verified that the capacity for spontaneous breathing has been lost, and it can be relied on because ongoing spontaneous breathing requires a functioning brain.

The British have always misunderstood their own view in the same way. They identified and still identify death with brainstem (rather than total brain) failure because the capacity for consciousness and the capacity for spontaneous breathing are supposed to be brainstem functions [13, 61, 64]. But although respiration is generated in the brainstem, the capacity for breathing is, of course, not dependent only on the brainstem. When respiration and circulation stop because of the failing functioning of a person’s heart or lungs, and she is not ventilated, her brainstem will normally be destroyed within seconds or minutes; but that very causal sequence shows that loss of brainstem function is not itself the basic standard, only a reliable diagnostic one. If cessation of spontaneous respiration and circulation is truly irreversible in a given case, and the individual in question has also already irreversibly lost the capacity for consciousness, she is already dead during those seconds or minutes. It may never be possible to identify such a case, but that does not undermine the conceptual point.

## The meaning of irreversibility

The groundwork has now been laid for discussing the basic question raised above. If the moment of death is determined by applying these basic criteria—namely, cessation of the capacities for consciousness and spontaneous respiration—can a DCD donation start when they have been met? Or is any medically acceptable waiting period too short?

A first point of criticism is straightforward: after waiting five minutes, or even substantially longer, the loss of the relevant respiratory and circulatory functions is not irreversible, for it is possible to restart mechanical ventilation; and in some (perhaps many) cases, respiratory and circulatory functions would then be resumed so that all of the other dependent functions on Shewmon’s list could then resume as well [55, 65–72]. What defenders of the compatibility of DCD donation and the DDR have done so far, almost invariably, is concede this point. Yes, they agree, the loss of relevant functions is not irreversible, strictly speaking. But, they suggest, one should in this case be content with a weaker meaning of “irreversible,” as equivalent to “permanent.” This proposal comes in two forms.

According to the first version—originally proposed in [73, 74]—the process *could* be reversed, but it *will* not be, since in stopping mechanical ventilation or attempts to reanimate, the doctor has already declared her intention not to do so (see further [16, pp. 45‒46; 61; 75–78]). This is a highly problematic proposal [69, 79]. It would mean that of two patients in exactly the same physical condition, one is dead while the other (say a patient in Japan or in an Orthodox Jewish hospital in New Jersey) is not, due to differences in their doctors’ plans.^20^ Recall that the DDR is supposed to protect people against the possibility that doctors will attempt to remove their organs prematurely; doctors must wait until a person is dead. But on this interpretation of the rule, whether or not one is dead *depends* on the doctor’s intentions! And consider the second basic criterion, the “irreversible” loss of consciousness. Would it make sense to say that a person who is dependent on artificial ventilation and defibrillation because of a spinal cord injury is dead when his doctor sedates him and intends to keep him in that state indefinitely? Suppose that a doctor makes a mistake in the dosage of midazolam she administers, with the result that the patient suddenly opens his eyes and asks her, “Doctor, am I dead already?” Should she say, “Yes, definitely, you are not supposed to talk”? Obviously, what matters is not the doctor’s intentions but the patient’s capacities, not what one *would* do but what one *could* do.

According to the second version, one should already start relying on normative considerations: the process *could* be reversed, but it *should* not be. Ventilation has presumably been stopped for good reasons, so that decision could not be reversed without violating ethical and legal norms [8, 80–84]. By restarting ventilation, the doctor would be either acting without the permission of the patient or his surrogate or acting without proper medical justification, or both. This version of the proposal is problematic in similar ways to the first one. Suppose continuation of ventilation is thought to be warranted in the case of a pregnant woman. How can it be that she is alive, while another woman who is not pregnant but otherwise in the same physiological state is dead? Suppose someone violates the relevant norm and restarts ventilation, with the result that respiration and circulation are restored. Should the organism still be called dead?^21^ Or should this be considered a case of resurrection from the dead? Both ideas are equally hard to swallow.

In any case, it is not a possible explanation for an “irreversible” process that one *will* not or *should* not reverse it. On the contrary, neither the intention nor the prohibition makes any sense if the process is truly irreversible. As Don Marquis asks [69, p. 27], is a gold ring insoluble in *aqua regia* because it would be wrong to dissolve it? Is fine china not fragile because everyone firmly intends not to break it? It should be kept in mind that irreversibility belongs to the core meaning of the concept of death; it does not belong to the open area of indeterminacy to be filled in by stipulation, possibly on normative grounds. It is no wonder that this kind of defense elicits accusations of dishonesty: If the public is told that the loss of functions should be irreversible, but “irreversible” means “permanent” in either of the two senses that I have discussed, then the DDR is only being paid lip service. It has been pointed out that a permanent cessation of circulation inevitably eventuates in a truly irreversible one,^22^ but that insight leads only to the inescapable conclusion that one still has to wait a bit longer. To assert that something is the case is essentially different from predicting that it will be [88, p. 106].

And yet all this verbal maneuvering or gerrymandering is unnecessary. Remember that in addition to this first requirement of irreversible loss of the capacity for consciousness, the second criterion for death laid out above is irreversible loss of the capacity for *spontaneous* respiration. So the fact that respiration can be restored by restarting ventilation is neither here nor there; it would be relevant only if after a while the ventilator could be stopped without cessation of these functions following immediately.^23^

In an important paper from 2010, Shewmon, diverging from his former position, suggests that our concept of death is to some extent ambiguous and can be refined in either of two ways, yielding two different concepts with separate contexts of use [32]. One of those concepts (“deanimation”) is an ontological one—namely, the irreversible cessation of consciousness and of the circulation of oxygenated blood, whether supported or unsupported [32, p. 281]. The other concept (“passing away”) is a sociolegal one, used in the medical practice of determining death—namely, the permanent cessation of consciousness and of the circulation of oxygenated blood [32, p. 278].^24^

By recognizing that the concept we have inherited from our ancestors, and still actually use, is ambiguous and in need of refinement, Shewmon in effect acknowledges the concept’s indeterminacy. It is to be applauded that his account does not understand “permanence” to be a possible meaning of “irreversibility”—on Shewmon’s view, permanence and irreversibility characterize two different refinements of the concept of death. However, both of these refinements are problematic. With respect to the ontological one, Shewmon concedes that there are very few contexts in which the concept is actually needed. But if it is not an explication of the actual concept of death, why muddy the waters with such stipulations? Why is it not enough to speak of the irreversible cessation of consciousness and the circulation of oxygenated blood on those few occasions where this is what people want to talk about? Proliferating senses of “death” will only increase confusion unnecessarily. But the one determinate concept that we really need cannot be the sociolegal one Shewmon proposes either, precisely because it focuses on permanence instead of irreversibility. Shewmon is aware of the fact that whether someone is “dead” in this sense depends not only on her physical state but also on other people’s future decisions. This is a possibility that nobody who has made death declarations would ever entertain—divine interventions excepted.

If the law adopted “permanent cessation” as the defining criterion of death (or even an adequate operational standard),^25^ that would, as critics of the whole-brain criterion allege, amount to use of a legal fiction.^26^ The law, however, cannot acknowledge that its understanding of death is a mere fiction, for that would evacuate the dead donor rule of all meaning, and this rule lies at the foundation of the law everywhere—whether stated explicitly or not. One cannot assure people in good faith that organs will be harvested only from dead bodies if one accepts that living bodies are declared dead at the moment when doctors are allowed to harvest those organs. The statement that organs will be taken only from dead bodies is, then, a mere tautology. If this is a correct understanding of the actual medical practice and its legal endorsement, it is also highly questionable whether consent for organ donation, as it is normally given, is sufficiently informed to be valid [48, 55].

On the view I have defended, however, the only fictive element in the law is that the term “death” is, for good reasons, given a determinate content that it lacks outside the legal context. That is quite normal for the use of legal concepts.

## Irreversibility and certainty

So we should concentrate on the capacity for spontaneous breathing. A second criticism of existing DCD protocols is that the waiting periods they prescribe are insufficient for fully excluding the possibility of the return of that capacity, however temporary. Until recently it was thought that the longest period of time between a determination of death on circulatory criteria and autoresuscitation ever observed was sixty-five seconds. This finding justified restricting the waiting period in some American and Australian hospitals to only two minutes. A 2010 systematic review of all known reports of autoresuscitation published through July 2008, however, corrected this finding [96] (confirmed by [84]). It turns out that all of these cases involve autoresuscitation after failed attempts to reanimate the patient; there were no reported cases of autoresuscitation when cessation of respiration occurred after the withdrawal of treatment. The reported time between the determination of death and autoresuscitation ranged from a few seconds to thirty-three minutes [96, p. 1248], but the methods of monitoring and estimating times were largely unreliable. In the most reliably reported cases, with continuous electrocardiogram monitoring, autoresuscitation occurred up to seven minutes after the reanimation attempts had ceased. A more recent study reports cases of autoresuscitation where cessation of respiration occurred after the withdrawal of treatment but only in the first minute of the no-touch period [8]. The total number of observations (eight in the 2010 survey) is very limited to begin with. Hence no doctor would be shocked into believing in miracles if at some point autoresuscitation were observed after more than seven minutes or after more than one minute of no-touch once the ventilator is turned off. If it is acknowledged that such a case is possible, it cannot at the same time be said that reversal of the loss of the relevant functions is impossible—so the criticism goes [67; 94, pp. 552–556].

All of this is all the more worrying given that the same reasoning applies to the loss of consciousness. One cannot fully exclude in any given instance the possibility that, at the moment of autoresuscitation, the patient’s brain is somewhat less devastated than is normally the case when spontaneous circulatory and respiratory functions stop (and poisoning, use of drugs, and so forth are excluded as possible causes). In such cases, some remnants of mental activity could also still be present. The patient could then even remain in that condition of prolonged mental activity for some time when ventilation is restarted [72]. And if autoresuscitation is still possible after five minutes of no-touch, it would seem that it is not strictly impossible that the brain at that moment still produces some flickerings of awareness. We really do not know how much time it takes before one can be absolutely sure that the capacity for consciousness has been irreversibly lost [97].^27^

This line of argument presupposes that a judgment of irreversibility requires certainty. But is this assumption correct? What, exactly, does it mean to say that a patient’s loss of functions “cannot” be reversed? Clearly it does not mean that such a reversal is conceptually impossible—if a person claims to have witnessed a miracle of resurrection, one may be fairly sure that the claim is false, but one cannot reject it as absurd a priori [53].^28^ But it also does not mean that the reversal is epistemically impossible, that it contradicts something known to be true with absolute certainty. If someone says, “It might be irreversible, but I am not sure,” I cannot reply, “But in that case reversal *is* possible, so you really *are* sure (that the claim is false).” The claim is not that reversal is impossible in that sense. After all, people way back when are known to have made dramatic mistakes in their assessments of death on account of a patient’s chest movements or his pulse. But from the fact that some judgments of irreversibility were mistaken it does not follow that all of them were mistaken.

What it means when someone says that a patient’s loss of functions is irreversible, I propose, is the following: given the condition of the patient (as it really is, not as it is thought to be) and the actually available technological means, reversing the loss would be incompatible with the laws of nature (as they really are, not as they are believed to be).^29^ This is a claim about the facts, not about one’s knowledge of the facts. It follows that it would not be inconsistent to say, “The loss cannot be reversed, but no, I am not 100% certain that reversal is not possible.” Saying that the loss is irreversible is still not equivalent to saying that the loss is permanent. Even if one thinks it merely possible or improbable that the loss is irreversible, what one thinks possible or improbable is still that it cannot be reversed. Irreversibility is a theoretical or ontological concept, as permanence is; certainty is an epistemological one.

So it is an open question how sure we have to be that the loss of functions is irreversible, in the sense I have explained. The answer is, of course, very sure—not because otherwise the claim that the loss is irreversible is false *ipso facto*, but because the DDR would lose its point if we accepted anything less. But it seems that this requirement is in fact satisfied after a waiting period of, say, five minutes—most clearly so in cases of controlled or expected DCD.^30^ As for the possibility of remaining consciousness, everything currently known about the development of the condition of the typical DCD donor suggests that this possibility, if it exists at all, is extremely low. First comes a head trauma, stroke, or cardiac arrest followed by cerebral anoxia, the very need for artificial support of breathing already indicating very serious initial brain injury. Next comes a cascade of ever-increasing damage to the brain, caused by a vicious cycle of edema and deprivation of oxygen, with the result that signs of consciousness do not return and cessation of breathing follows when the ventilator is turned off. Such patients are already close to satisfying the requirements of total brain failure. It is not necessarily in contradiction with this assessment to forbid reperfusion of the brain, or even to use anesthesia, to make sure that any remaining error, however small, is on the side of safety. After all, whenever a dying patient is deeply and permanently sedated, analgesics are routinely used in addition to sedative drugs, but this is not because the depth of the patient’s coma is actually doubted. If analgesics are being used, it may still be strictly *possible*, both conceptually and epistemically, that the patient does not meet the aforesaid criteria for the determination of death, by virtue of his having either the capacity for conscious experience or the capacity for spontaneous breathing for several more seconds or minutes. But the probability is extremely small, and there is hardly harm involved in accepting it.

This is, for sure, a normative argument, as is the argument that the DDR would lose its point if greater uncertainty were allowed. But such normative arguments are proper ones at this stage because they are not used for twisting the meaning of “irreversibility” but for determining the acceptable level of uncertainty for an assessment of irreversibility.

The great disadvantage of the DCD procedure has always been the deterioration of the organs due to warm ischemia from the moment that the ventilator has been decoupled onward. In recent years, a standard reanimation technique, extracorporeal membrane oxygenation (ECMO), has been used to solve this problem by providing oxygen to the body through arterial and venous catheters inserted before ventilation is discontinued—thereby preventing warm ischemia and retaining the quality of the organs. But by providing oxygen to the organs, including the brain, not only before but also after the beginning of asystole, the rationale for a waiting time is undermined, since reperfusion may prevent completion of the ischemic destruction of the brain.^31^ So if this technique is allowed, one could just as well skip the waiting period. How serious is this consequence? Remember that normally the very fact of the cessation of spontaneous respiration and circulation strongly indicates a condition close to total brain failure, and hence close to loss of the capacity for consciousness and for spontaneous breathing. This consideration is all the more weighty when the body, before respiration and circulation stop, already has an alternative source of oxygen, as is provided by ECMO. Thus, one can still say that it is highly probable that the two relevant capacities have been irreversibly lost, although the use of ECMO certainly decreases this probability. This decrease in probability may present a good reason for prohibiting the use of this technique. But the reason for this prohibition would not be that the loss of relevant capacities has turned out to be reversible, just that there needs to be more certainty that it is irreversible. Theoretical and epistemic claims should not be conflated.

## Conclusion

Have I succeeded in showing that DCD protocols may be compatible with the DDR? I have argued that there are good reasons for the law to accept irreversible loss of the capacity for consciousness and irreversible loss of the capacity for spontaneous respiration as the proper criteria for determining death, but it could be objected that if criteria must be stipulated in order to achieve compatibility, this shows only that compatibility in a robust sense has not been achieved. However, I have argued, on the one hand, that there is no more robust sense of compatibility to be sought because there is no fully determinate truth as regards the definition of death and, on the other hand, that the law is interested in maintaining the DDR so as to protect people from a declaration that they might consider premature. It matters, then, that almost no one cares to be “kept alive” (in some extended sense) when the capacity for consciousness and the capacity for spontaneous breathing have been lost forever. Therefore, the compatibility I have shown to be possible is ultimately all the compatibility that one can hope for.

## Footnotes

1 This used to be called NHB, non-heart-beating donation.

2 In 2000, of patients who died in intensive care, 3.2% were declared brain dead in Northern Europe, while 12.4% were declared brain dead in Southern Europe [5].

3 This is because severe hypotension is a confounder and should be corrected before conducting a brain failure assessment.

4 The Austrian and Czech protocols require ten minutes; the Italian protocol even requires twenty [7]. For the Australian and United States protocols, which require only two minutes, see [8].

5 Though similar in intent, this is different from allowing people to choose their own definition of death, as Robert Veatch and Lainie Ross [10] propose.

6 See Michael Nair-Collins et al. [11] for a more optimistic estimation.

7 Cf. [13; 14; 15, pp. 11–13; 16, p. 40; 17, pp. 58ff], majority view; [18], minority view.

8 Both basic criteria and derivative, diagnostic ones should be distinguished, on the one hand, from specific medical tests for applying such standards (e.g., unresponsiveness, lack of cranial nerve reflexes, a flat electroencephalogram, or apnea) and, on the other hand, from a metaphysical account of the nature of death. The usual tripartite scheme, introduced by James Bernat and colleagues [21], is therefore inadequate. The President’s Commission [20, pp. 55–56] adopted instead the four-part scheme (conception of death, basic criteria, diagnostic standards, medical tests) originally proposed by Alexander Capron and Leon Kass [22].

9 See footnote 11.

10 The best evidence for this comes from cases of brain death in pregnant women, where fetal support has been maintained for up to 107 days [17, 27, 28]. Susan Repertinger and colleagues [29] report a case of two-decades-long “survival” after brain death.

11 If it is merely said that a person with no brain activity is on mechanical support that keeps his heart and lungs going, the overwhelming majority of respondents (86.2%) would classify that person as dead [34].

12 A similar view is that the concepts of “life” and “death” apply to instances which have only a family resemblance to each other, and hence cannot be defined in terms of necessary and sufficient conditions [41]. However, some of the conditions on Winston Chiong’s list are clearly sufficient ones for life (consciousness and spontaneous breathing), and all of them depend on continuous respiratory function, whether supported or unsupported. For other related views, see [42, 43]. The claim that the concept of death is indeterminate should not be confused with the view that it does not refer to a moment but to a process. As per Kass, dying is a process, but it ends in the state of death (quoted in [44, p. 84]).

13 According to the account given by Maureen Condic [45], the ongoing functions listed by Shewmon are examples of mere coordination: a stimulus bringing a range of cells either to a single type of response or to a set of synchronic responses. True integration uses information from multiple sources to achieve a set of responses that work together for the continued healthy functioning of the whole organism, and this requires a functioning brain (see also [46]; and [18, pp. 72–83], majority view). Even if this is correct (cf. [37]), it does not answer the question of why the higher level of organization is required to consider a human organism alive.

14 The white paper does not present itself as a rival account, as is suggested in [45, p. 275, note 3; 47; 48]. For an older view that integrates the accounts, see [23] (cf. [49]).

15 See [18, pp. 93–94], minority view; [26; 51, 52, p. 176; 53–55].

16 Both Condic [45] and Melissa Moschella [56] appeal to the essential nature of the human being as a rational animal to support their claim that persistence of any mental function and “persistence of global, autonomous integration of vital functions” [56, p. 8] are both sufficient to show the presence of a living human being. But if *homo sapiens* is essentially a rational animal, it seems to follow that rationality and animality are both necessary, not sufficient, conditions.

17 Such higher-brain conceptions of death do not merely specify the concept where it is indeterminate; they change its determinate borders.

18 For a survey of views within world religions, see [57]. There may be other reasons to take account of such views in the law. The New Jersey Declaration of Death Act states that death shall not be declared on the basis of neurological criteria if such a declaration would violate the personal religious beliefs of the individual [58, p. 291].

19 Cf. [43, pp. 34–36]. What about a fetus in the first weeks of its brain development [43, pp. 19, 37; 59]? Is it not a living human being even if it still lacks capacities for consciousness and spontaneous breathing? Perhaps it should be considered a living part of a larger living organism [60].

20 This is a point acknowledged but accepted by Shewmon [32] and Dieter Birnbacher [43, p. 41].

21 Calixto Machado and Julius Korein [85] raise this question without noticing how devastating it is for their own interpretation of irreversibility.

22 Without intervention, permanence quickly develops into irreversibility; therefore, “permanent cessation serves as a valid stand-in for irreversible cessation” [86, p. 965] (cf. [87]).

23 During the first seconds or minutes of asystole, it is possible to artificially restore the blood supply to the brain in order to minimize ischemic damage. However, doing so could potentially restore some cerebral functioning. For this reason, there is consensus at this point that such asystolic provision of blood supply to the brain should be prohibited. In any case, this possibility of restoration means that during these first seconds or minutes of asystole, the loss of function is to be considered permanent but not irreversible. It should therefore be said that death is determined at the end of the no-touch period, not at the beginning as some protocols say (see [7]). The Dutch *Wet op de Orgaandonatie* [9, art. 22 lid 3] is confused when it requires a no-touch period from the moment of “irreversible cardiac arrest” onward [16, pp. 95–98].

24 For a somewhat similar proposal by Bernat, who was, for a time, the leading proponent of “permanent” as a possible understanding of “irreversible,” see [46, 89]. His position differs from Shewmon’s in that he does not claim that the standard used in the medical practice of determining death rests on a possible understanding of the conventional meaning of “death.” As a result his position is practically indistinguishable from the view of those critics of the whole-brain conception of death who believe that that conception is a useful legal fiction, see next footnote. On Bernat’s view, it is primarily a conventional fiction of medicine.

25 Even in that case, some statutes would have to be changed, because they contain a reference to irreversibility (e.g., [9]).

26 The use of a legal fiction has been defended in [90–95].

27 This is why DCD is still legally forbidden in Germany. However, no set of diagnostic tests can make it absolutely certain that all functions of the entire brain have ceased.

28 Because he interprets the “cannot” of “irreversible” as conceptual, David Cole mistakenly concludes that irreversibility is not a core element of the meaning of death [73].

29 David Oderberg objects that on this analysis, of two people in exactly the same condition, one would be dead (in 1650) and the other would be alive (in 2018) [59]. On his view, a body is certainly dead only if it has begun to decompose. But a future technology might (in 2400) be able to reverse the decomposition of the body in its early phases.

30 Bernat and colleagues [98] propose seven minutes of no-touch in the cases of unexpected DCD, but in most countries in which that practice has been introduced, five minutes is still considered sufficient [16, p. 83].

31 It would be possible to prevent this undesired consequence by blocking the delivery of oxygen to the brain at the diaphragm. But this can be done only using an invasive procedure, and, as the 2008 President’s Council on Bioethics  rightly observes, it cannot be said consistently that the patient is already beyond harm at that stage [17].
